# The Effects of Meldonium on the Renal Acute Ischemia/Reperfusion Injury in Rats

**DOI:** 10.3390/ijms20225747

**Published:** 2019-11-15

**Authors:** Siniša Đurašević, Maja Stojković, Ljiljana Bogdanović, Slađan Pavlović, Slavica Borković-Mitić, Ilijana Grigorov, Desanka Bogojević, Nebojša Jasnić, Tomislav Tosti, Saša Đurović, Jelena Đorđević, Zoran Todorović

**Affiliations:** 1 Faculty of Biology, University of Belgrade, 11158 Belgrade, Serbia; jasnicn@bio.bg.ac.rs (N.J.); jelenadj@bio.bg.ac.rs (J.Đ.); 2 School of Medicine, University of Belgrade, 11129 Belgrade, Serbia; maja.stojkovic@med.bg.ac.rs (M.S.); ljbogdanovic77@yahoo.com (L.B.); zoran.todorovic@med.bg.ac.rs (Z.T.); 3 Institute for Biological Research “Siniša Stanković”–National Institute of Republic of Serbia, University of Belgrade, 11060 Belgrade, Serbia; sladjan@ibiss.bg.ac.rs (S.P.); borkos@ibiss.bg.ac.rs (S.B.-M.); iligri@ibiss.bg.ac.rs (I.G.); dekana@ibiss.bg.ac.rs (D.B.); 4 Faculty of Chemistry, University of Belgrade, 11158 Belgrade, Serbia; tosti@chem.bg.ac.rs; 5 Institute of General and Physical Chemistry, University of Belgrade, 11158 Belgrade, Serbia; sasatfns@uns.ac.rs; 6 University Medical Centre “Bežanijska kosa”, University of Belgrade, 11080 Belgrade, Serbia

**Keywords:** ischemia/reperfusion, kidney, inflammation, oxidative stress, antioxidative defence, noradrenaline, lipidomics, rats

## Abstract

Acute renal ischemia/reperfusion (I/R) injury is a clinical condition that is challenging to treat. Meldonium is an anti-ischemic agent that shifts energy production from fatty acid oxidation to less oxygen-consuming glycolysis. Thus, in this study we investigated the effects of a four-week meldonium pre-treatment (300 mg/kg b.m./day) on acute renal I/R in male rats (Wistar strain). Our results showed that meldonium decreased animal body mass gain, food and water intake, and carnitine, glucose, and lactic acid kidney content. In kidneys of animals subjected to I/R, meldonium increased phosphorylation of mitogen-activated protein kinase p38 and protein kinase B, and increased the expression of nuclear factor erythroid 2-related factor 2 and haeme oxygenase 1, causing manganese superoxide dismutase expression and activity to increase, as well as lipid peroxidation, cooper-zinc superoxide dismutase, glutathione peroxidase, and glutathione reductase activities to decrease. By decreasing the kidney Bax/Bcl2 expression ratio and kidney and serum high mobility group box 1 protein content, meldonium reduced apoptotic and necrotic events in I/R, as confirmed by kidney histology. Meldonium increased adrenal noradrenaline content and serum, adrenal, hepatic, and renal ascorbic/dehydroascorbic acid ratio, which caused complex changes in renal lipidomics. Taken together, our results have confirmed that meldonium pre-treatment protects against I/R-induced oxidative stress and apoptosis/necrosis.

## 1. Introduction

Acute renal ischemia/reperfusion (I/R) is a temporary restriction of kidney blood supply, followed by blood flow restoration and re-oxygenation. As the main cause of acute kidney injury, it may occur due to aortic dissection, surgery, infarction, sepsis, or organ transplantation [1]. Due to mortality being as high as 50% and the lack of efficient pharmacotherapy, I/R is still a challenging condition in clinical medicine [2,3]. Although the precise pathophysiology of renal I/R is unclear, multiple pathways are involved, such as reactive oxygen species (ROS) formation, inflammation, apoptosis, necrosis, and signalling pathway disruption [4,5].

Meldonium is an anti-ischemic drug clinically used to treat myocardial and cerebral ischemia [6]. Meldonium inhibits gamma-butyrobetaine dioxygenase, the enzyme catalyzing the final step in the carnitine biosynthesis, and the carnitine palmitoyltransferase-1, an inner mitochondria membrane enzyme that catalyzes transfer of the acyl group from coenzyme-A to carnitine. As a result, long-chain fatty acids transport from cytosol into mitochondria is reduced and redirected to peroxisomes [7]. In peroxisomes, long-chain fatty acids are metabolized to medium- and short-chain acyl carnitines for further oxidation in mitochondria, which prevents the mitochondrial accumulation of toxic long-chain intermediates [8]. In this way, meldonium decreases the risk of a long-chain fatty acid and metabolism mediated mitochondrial injury, thus shifting energy production from fatty acids oxidation to less oxygen-demanding glycolysis, which is more favorable under ischemic conditions.

To address this issue, the aim of the present experiment was to investigate the effects of four-week meldonium pre-treatment with 300 mg/kg b.m./day of rats subjected to a well-established experimental model of renal I/R, with ischemia lasting for 45 minutes, followed by 4 hours of reperfusion [9]. The changes in body mass, body mass gain, and food and water intake were measured in sham and I/R operated animals, with or without meldonium pre-treatment. The kidney concentration of carnitine, glucose, and lactic acid were measured, as general markers of meldonium metabolic action. The degree of kidney apoptosis and necrosis was evaluated by measuring kidney Bax/Bcl-2 ratio [10], as well as serum and kidney levels of high mobility group box 1 protein (HMGB1) [11] together with the kidney histology analysis [12]. The kidney antioxidative defense was investigated by measuring the tissue expression of the nuclear factor erythroid 2-related factor 2 (Nrf2), haeme oxygenase 1 (HO-1), manganese superoxide dismutase (MnSOD), phosphorylation ratio of protein kinase B (pAkt/Akt), mitogen-activated protein kinase p38 (pp38/p38), cooper-zinc superoxide dismutase (CuZnSOD) activity, catalase (CAT), glutathione peroxidase (GSH-Px), glutathione reductase (GR), glutathione S-transferase (GST), free sulfhydryl group (SH) concentration, and lipid peroxidation level (thiobarbituric acid reactive substances, TBARS) [13,14,15]. Since ascorbic acid is a well-known effector of the antioxidative system [16], our experiments also included a determination of ascorbic acid (AA) and dehydroascorbic acid (DHA) ratio (AA/DHA) in the rat serum, adrenal gland, liver, and kidney as a measure of its activity. Moreover, the link between ascorbic acid and catecholamine production in the adrenal glands is also well known [17,18]. Keeping that in mind, together with the fact that performed surgical interventions represent the stress that engages the sympathoadrenal system, we also determined the noradrenaline (NA) and adrenaline (AD) concentrations in adrenal glands. The analysis of 13 fatty acids was performed to evaluate the effects of meldonium and I/R on kidney lipidomics.

## 2. Results and Discussion

There is little literature on the effects of meldonium on body mass gain and food and water intake, except for two studies. Porter et al. [7] showed that a 10-day meldonium supplementation did not alter these parameters. Liepinsh et al. [19] showed that meldonium decreased body mass gain in Zucker obese rats, but only in combination with metformin.

**Figure 1:**
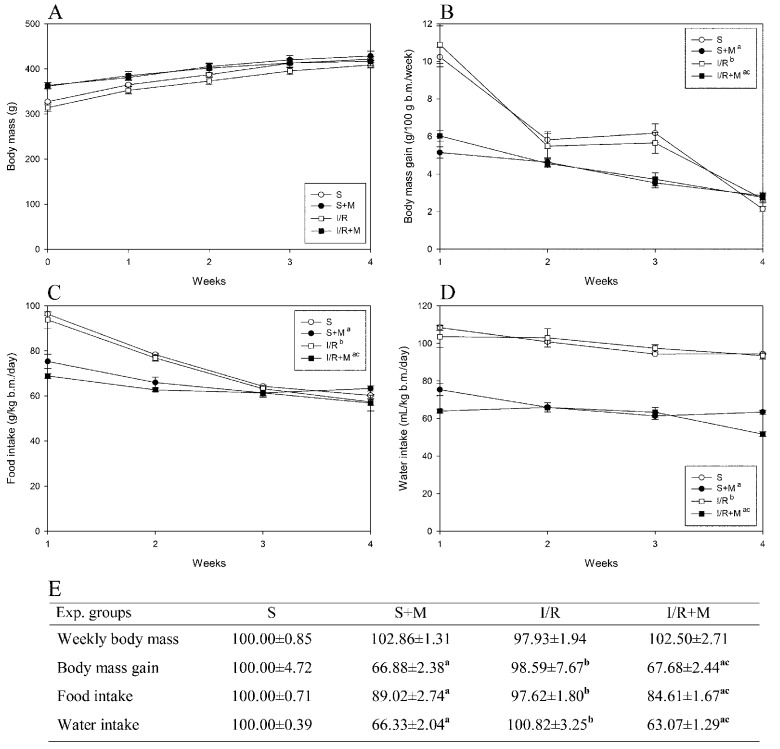
Time-course curves of: (**A**) weekly body mass (g); (**B**) body mass gain (g/100 g b.m./week); (**C**) food intake (g/kg b.m./day); and (**D**) water intake (mL/kg b.m./day, **E**): the area under a curve values (% of sham) of weekly body mass, body mass gain, and food intake and water intake. Group categories abbreviations: S—sham operated rat group; S + M—sham operated + meldonium rat group; I/R—ischemia/reperfusion rat group; I/R + M—ischemia/reperfusion + meldonium rat group. Number of animals per experimental group: *n* = 8. The data are given as mean ± standard error. Minimal significant level: *p* < 0.05. Significantly different: **^a^** in respect to S; **^b^** in respect to S + M; **^c^** in respect to I/R group.

Our measurement of body mass and food and water intake were completed before the surgical procedures (sham or IR), meaning that all detected changes were caused by meldonium and not by surgical procedures. As can be seen from our results, meldonium decreased body mass gain (Figure 1B,E) and food (Figure 1C,E) and water intake (Figure 1D,E) in sham and I/R operated animals in comparison with their respecting controls. The skin pinch test we performed showed no animal dehydration and the trend of body mass changes in all experimental groups was similar, as proven by the curves with the similar slope running parallel to each other (Figure 1A). We assume that meldonium, by shifting mitochondrial ATP synthesis from fatty acid oxidation to less oxygen-consuming glycolysis [8], leads to a more efficient yield of energy and metabolic water [20,21], which thus reduces food and water intake. Bearing in mind that a high-fat diet induces obesity in rats [22], and that meldonium modulates cellular energy production pathway [8], it may be concluded that meldonium has the ability to act as an anti-obesity agent.

The evidence that meldonium actually shifts metabolism towards a better utilized glucose are that there are changes in the kidney content of carnitine, glucose, and lactic acid (Table 1). While I/R did not change kidney concentration of carnitine and glucose, it caused a 3.6-fold increase in lactate concentration, which is in line with the literature that confirms hypoperfusion as the main source of tissue lactate concentration increase [23,24]. In aerobic conditions, the end product of glycolysis is pyruvate, which upon formation enters the Krebs cycle to avoid lactate production [25]. Under anaerobic conditions, the end product of glycolysis is lactate, so any obstruction in tissue oxygen supply, as is the case with I/R, leads to its tissue concentration increase [26]. The I/R-induced kidney lactate concentration increase was reduced by meldonium for almost 60%; the same effect was present in sham operated animals (26.6-fold decrease in comparison to untreated rats). It should be noted that in sham and I/R operated animals, meldonium decreased carnitine and glucose tissue concentration (Table 1), which his consistent with the literature data that shows an increase in glucose uptake and decrease in lactate concentration in mice hearts treated with meldonium [27]. In this sense, it can be said that our results confirm that meldonium stimulates aerobic oxidation of glucose by the inhibition of carnitine synthesis, as previously suggested by Asaka et al. [28].

During I/R, depleted oxygen supply causes dysfunctionality of the ATPase-dependent ion transport and intracellular ATP. Further, pH levels decrease, intracellular and mitochondrial calcium levels increase, and there is a disturbance in numerous signaling pathways [29]. Upon reperfusion, a restoration of normal oxygen level causes a rise in ROS generation, a release of cytokines and chemokines from activated tissue-resident macrophages, and infiltration of pro-inflammatory neutrophils into ischemic tissues [23]. All these changes lead to cell swelling and rupture, as well as consequent necrotic or apoptotic cell death. Cell death assumes a central role in the etiology of kidney pathologies after I/R [30]. Renal cells may die during ischemia itself, in which case early membrane damage leads to activation of multiple degradative systems in an uncontrolled fashion, referred to as necrotic. However, the reperfusion-induced increase in ROS generation contributes to abnormal signal transduction, cellular dysfunction, and cell death by apoptosis [31]. Cellular mechanisms that result in apoptosis represent a cascade of numerous amplifying steps, including activation of pro-apoptotic Bax protein, a member of the Bcl2 protein family [32]. It has been suggested that a delicate interplay between anti- and pro-apoptotic members of the Bcl2 family is necessary for the repair of the damaged renal cells after an ischemic insult [33]. Moreover, it has been reported that the over expression of anti-apoptotic Bcl2 can block both apoptosis and necrosis [32], as well as protect ischemic tissue against I/R-induced oxidative stress [34].

**Figure 2:**
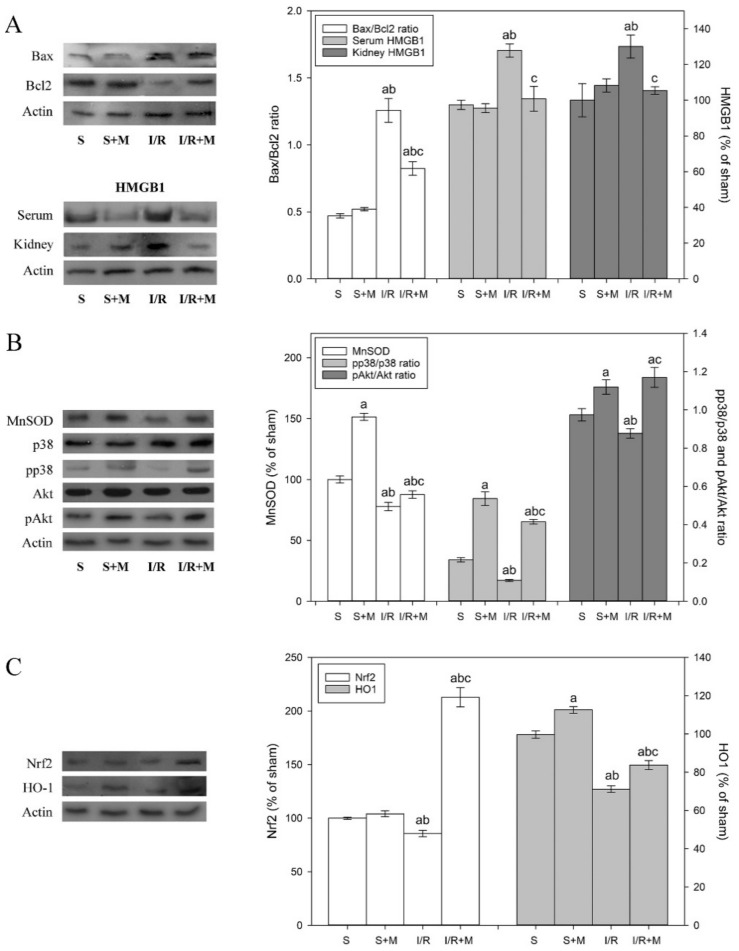
Representative immunoblots of protein expression levels: (**A**) kidney Bax, Bcl-2, HMGB1, and serum HMGB1; (**B**) kidney MnSOD and total and phosphorylated p38/Akt; and (**C**) kidney Nrf2 and HO-1. β actin was used as a loading control. Group categories abbreviations: S—sham operated rat group; S + M—sham operated + meldonium rat group; I/R—ischemia/reperfusion rat group; I/R + M—ischemia/reperfusion + meldonium rat group. Number of animals per experimental group: *n* = 8. Data are given as mean ± standard error. Minimal significant level: *p* < 0.05. Significantly different: **^a^** in respect to S; **^b^** in respect to S + M; **^c^** in respect to I/R group.

To examine the effect of meldonium pre-treatment on the extent of apoptosis activation in the kidney subjected to I/R, we measured the changes in the Bax/Bcl2 ratio. Our results showed that I/R increased the Bax/Bcl2 ratio by 2.7-fold and that meldonium reduced this increase for 35% (Figure 2A). This finding indicates that the acceleration of pro-apoptotic events during I/R—as well as its effective reduction by meldonium—is partially similar to the work of Shen et al. [10], who showed that ischemic preconditioning decreased the Bax/Bcl2 ratio and increased under I/R conditions.

**Figure 3:**
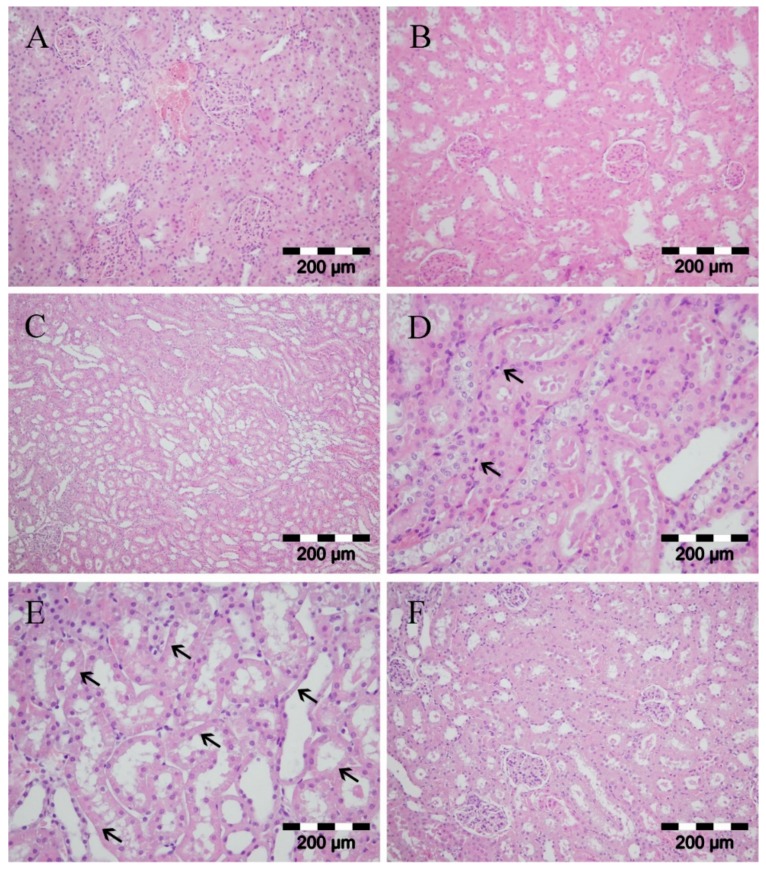
Kidney histology analysis. Group categories abbreviations: (**A**) sham operated rat group; (**B**) sham operated + meldonium rat group; (**C**–**E**) ischemia/reperfusion rat group; (**F**) ischemia/reperfusion + meldonium rat group. (**A**,**B**) normal histological structure of glomeruli and tubules; (**C**) severe tubular necrosis with dilatation of the tubular structure; (**D**) tubular necrosis and cast formation, just rare cells showed apoptotic changes with dense nucleus and no inflammation (arrows); (**E**) the reduction or loss of brush border, dilatation of tubular lumina, necrosis of epithelial tubular cells as observed by the loss of nuclei (notice the loss of nuclei in many tubular cells marked by arrows); (**F**) moderate kidney damage, focal tubular necrosis, and moderate dilatation of the tubular structure.

Apoptotic and necrotic cell death occur simultaneously under I/R conditions. Cellular death due to necrosis result in the loss of cell membrane integrity and uncontrolled release of damage-associated molecular pattern molecules (DAMPs) into the extracellular space. HMGB1, which belongs to the DAMPs family, diffuses out of stressed, damaged, or dying cells, which is why it serves as necrotic marker [35]. Our results show that I/R and meldonium changed HMGB1 expression in a manner similar to Bax/Bcl2. While I/R caused an increase in serum (22%) and kidney (30%) levels of HMGB1, concurrent meldonium pre-treatment reduced them for 20% (Figure 3A). These findings proved that meldonium protects renal cells against I/R-induced necrosis, confirming results of Wu et al. [12], who showed that treating mice with anti-HMGB1 antibodies protects kidneys against I/R injury.

It is known that I/R elevates ROS generation and decreases antioxidant defense through antioxidative enzyme gene downregulation. Studies have reported the loss of the MnSOD enzyme activity following I/R, with the MnSOD inactivation prior to the onset of renal damage, which suggests an upstream of oxidative damage to renal damage [36]. It has also been reported that upregulation of mitogen-activated protein kinase p38 alleviates I/R-induced renal tissue injury by activating protein kinase B [37] and inducing Bcl2 and MnSOD expressions [38,39]. As can be seen from our results, while I/R decreased the kidney MnSOD activity, meldonium pre-treatment increased it in both sham and I/R operated animals (Table 2). The changes in MnSOD activity were accompanied by appropriate changes in MnSOD expression level, as well as in the pp38/p38 and pAkt/Akt ratios as a measure of their activation (Figure 2B). In comparison to sham operated animals, I/R decreased MnSOD expression level by 20% and pp38/p38 and pAkt/Akt ratios by 50% and 10%, respectively. On the contrary, in I/R animals, meldonium increased kidney expression of MnSOD by 1.12-fold and phosphorylation of p38 and Akt by 3.7- and 1.3-fold, respectively. Similarly, in kidney of sham operated animals, meldonium increased MnSOD expression level by 1.51-fold and p38 and Akt phosphorylation by 2.3- and 1.2-fold, as compared to S group rats. These results clearly indicate that I/R causes adverse changes in kidney antioxidative defence, while meldonium improves it in both I/R and sham operated animals.

We also investigated the protein expression of Nrf2, which has been shown to be the most important inducible transcription factor that exerts protective effects against renal ischemic damage by regulating the endogenous antioxidant system. Once activated in cytosol, Nrf2 trans-activates antioxidant response elements, which further promote the CuZnSOD, GSH-Px, GST, CAT, and HO-1 expression increase [40]. Several lines of evidence indicate that induced expression of HO-1 attenuates cellular damage and reduces apoptosis, while HO-1 inhibition aggravates it [41]. Our results showed that I/R decreased kidney Nrf2 (16%) and HO-1 (31%) expression levels by 16% and 31%, respectively, while concurrent meldonium pre-treatment increased them by 2.6-fold in the case of Nrf2, and 1.2-fold in case of HO-1 (Figure 2C). These findings suggest a new role of the Nrf2 signaling pathway in a meldonium protection against renal I/R injury.

Unexpectedly, changes in Nrf2 expression were not accompanied by appropriate changes in the activity of Nrf-2 associated antioxidant enzymes. As can be seen from Table 2, I/R did not change CuZnSOD, GSH-Px, GR, and GST activities in comparison to sham operated animals. At the same time, meldonium reduced CuZnSOD activity in rat kidneys from both the S + M and I/R + M groups, especially when compared to their respective controls and GSH-Px/GR activities in rats from the I/R + M groups and I/R rat group. In fact, the only enzyme whose activity somewhat accompanied changes in the Nrf2 expression was CAT. Its activity was reduced in the I/R group and unchanged in the I/R + M group. A possible explanation may be that the total Nrf2 transcriptional activity depends not only on its expression but also on availability of binding partners, the competition and/or cooperation with other activators and repressors, and crosstalk with other signaling pathways [42]. Moreover, a meldonium-induced increase in MnSOD activity assumes a more effective removal of superoxide anion radicals from the site of their most massive production, i.e., the mitochondrial respiratory chain. In this way, the propagation of oxidative stress from mitochondria to cytoplasm is prevented, which explains the decreased CuZnSOD activity in rat kidneys from the S + M and I/R + M groups, as well as the reduced GSH-Px/GR activity in animals from the I/R + M group. Actually, evidence that meldonium prevents I/R-induced oxidative stress are found from the changes in the concentration of thiol groups and in the level of lipid peroxidation (Table 2). The concentration of SH groups represents a degree of the cell reduced state, which is why its increase indicates reduced oxidative stress [43]. The level of lipid peroxidation, on the other hand, can be considered as a general indicator of oxidative stress. Our results showed that I/R decreases the SH group’s concentration and increases the level of lipid peroxidation, while concurrent meldonium pre-treatment decreases the level of lipid peroxidation in the I/R + M group in comparison to the I/R group, and increases the SH group concentration in both the S + M and I/R + M groups, especially when compared to their respective control.

We also investigated the ratio between ascorbic acid (the reduced form of vitamin C) and its oxidized form (dehydroascorbic acid). The AA redox reactions begin with a single-electron oxidation that generates a mono-dehydroascorbate radical (MDHA), which further dismutates DHA plus AA [16]. Both MDHA and DHA are reduced to ascorbate by several enzymatic systems in a process referred to as vitamin C recycling [44]. In this way, the AA/DHA ratio can be considered as an important element in evaluation of tissue antioxidative defense. Other than the kidney, the AA/DHA ratio was examined in several other tissues. The liver was selected because it is the site of vitamin C synthesis in rats [45]. Blood was selected because it is the major body depot of vitamin C and is a general marker of homeostasis. Adrenal glands were selected because of the exceptionally high content of vitamin C released into blood in a stressor-specific manner [46]. Moreover, adrenals are the site of catecholamine synthesis, in which vitamin C serves as a reducing equivalent [17] with the function to act as a cofactor of dopamine beta-hydroxylase during noradrenaline synthesis [47] and to keep easily oxidized catecholamines in a reduced state [18]. Since changes in the adrenal AA/DHA ratio may affect adrenal catecholamines content, we also measured NA and AD concentrations in adrenal glands.

As can be seen in Table 3, all examined tissues showed the same pattern of changes, while I/R decreased the AA/DHA ratio for 30–40%. Meldonium, alone or in combination with I/R, increased it. In the case of the I/R + M group, AA/DHA ratio values either return to or surpass the sham level. A decreased AA/DHA ratio represents a shift in the total vitamin C balance from the reduced into an oxidized form that indicates attenuation of tissue antioxidant defense and increased oxidative stress. Conversely, an increase in AA/DHA ratio indicates improved tissue antioxidant defense and thus a decrease in oxidative stress. These results confirm meldonium’s obvious yet unspecific tissue antioxidant action. This is in contrast to a previously described kidney-specific antioxidant action (Table 2, Figure 2).

However, it still remains uncertain how renal I/R, which we causes kidney oxidative stress (Table 2, Figure 2), may cause adverse changes to the AA/DHA ratio in distant organs (i.e., liver and adrenal glands) (Table 3). It is known that liver represents the major organ that can be remotely damaged by the renal I/R [48]. The renal I/R causes a release of the numerous inflammatory cytokines, activated leukocytes, and other neuro-humoral factors that ultimately affect the liver [49]. However, to the best of our knowledge, there is no literature on the similar effect of renal I/R on adrenal glands.

It is an interesting finding that I/R and meldonium only impact NA content in adrenal glands without changes in adrenaline content (Table 3). While I/R decreased rat adrenal NA content, meldonium increased it in rats from both S + M and I/R + M groups. Adrenal glands are known to have the ability to preferentially synthetize and secrete NA or AD. For example, insulin-induced hypoglycemia increases adrenaline secretion in cats, while hemorrhage or hypothermia preferentially increases that of noradrenaline [50]. The biological significance of this phenomenon is related to the different effects of NA and AD on target tissues, relative to different receptor subpopulations [51]. However, the biological significance in the adrenal content of catecholamines in the case of I/R and meldonium remains unclear.

Renal necrosis occurs secondary to ischemia [52]. Cellular mechanisms include the rapid loss of cytoskeletal integrity, the shedding of the proximal tubule brush, the cast formation from cells, debris detached from the basement membrane, and the leaking of glomerular filtrate from the tubular lumen to interstitium [53,54]. Our results showed no significant differences in histology between two sham operated groups (with or without meldonium pre-treatment) (Figure 3A,B). I/R produced severe tubular necrosis with dilatation of the tubular structure (Figure 3C), cast formation (Figure 3D), dilatation of tubular lumina, reduction or loss of brush border, and loss of a cells in some areas (Figure 3E). However, meldonium pre-treatment reduced histological evidence of renal I/R necrotic and apoptotic injury, especially regarding tubular structures (Figure 3F).

Lipids are fundamental components of biologic membranes. Depending on the chemical structure and biosynthesis, lipids can be divided into eight categories: fatty acids (FA), glycerolipids, sphingolipids, phospholipids, saccharolipids, sterol lipids, prenol lipids, and polyketides [55]. One of the most important lipid classes is fatty acids. With saturated or unsaturated straight-carbon chains—with lengths of 4–24 carbon atoms and 0–6 double bonds—fatty acids are a basic component of all lipids. Glycerolipids are composed of mono-, di-, and tri-esterified glycerols, which all differ in fatty acid content [56]. Many studies have shown that altered triglyceride synthesis and catabolism play important roles in the occurrence and development of numerous diseases [57].

To find whether meldonium alone or in combination with I/R will change the FA renal profile, we investigated the concentration of 13 fatty acids: myristic (tetradecanoic, C14:0), pentadecylic (pentadecanoic, C15:0), palmitic (hexadecenoic, C16:0), palmitoleic (cis-Δ9 hexadecenoic, C16:1), margaric (heptadecanoic, C17:0), stearic (octadecanoic, C18:0), oleic/elaidic (*cis* and *trans* Δ9-octadecenoic, C18:1 c + t), linoleic/linolelaidic (*cis* and *trans* Δ9,12-octadecenoic, C18:2 c + t), behenic (docosanoic, C22:0), cervonic (*cis*,*cis*-Δ13,16-docosadienoic, C22:6), and euric (*cis*,*cis*,*cis*,*cis*,*cis*,*cis*-Δ4,7,10,13,16,19-docosahexaenoic, C22:1). Under applied chromatographic conditions, cervonic and euric acids coelute at the same retention time, so their concentrations were calculated as the sum. The same was the case for *cis*- and *trans*- forms of C18:1 and C18:2, so the results were presented as the 10 fatty acids analysis (Table 4).

Our results showed that I/R, in most cases, caused an increase in FA concentration (C15:0, C16:1, C17:0, C18:1 c + t, C18:2 c + t, C22:0, and C22:6 + C22:1), with a decrease only in the case of C14:0 and C16:0, and no changes in the case of C18:0. Interestingly, changes caused by meldonium depend both on treatment and/or FA type. For example, in sham operated animals, meldonium generally increased concentration of almost all tested fatty acids, with the exception of C22:0 and C22:6 + C22:1, whose concentrations were not changed. However, in animals subjected to I/R, meldonium in some cases caused changes that corresponded to those in the S + M group, such as the concentration increase of C14:0, C16:0, C18:0, C18:1 c + t, and C18:2 c + t. This was not the case with C15:0, C16:1, and C17:0, whose concentrations meldonium decreased in the I/R + M group, which was in contrast to the increase it caused in S + M group. Similarly, in I/R animals meldonium caused a C22:0 and C22:6 + C22:1 concentration decrease, which was in contrast to sham operated animals where no chances were caused. 

**Figure 4:**
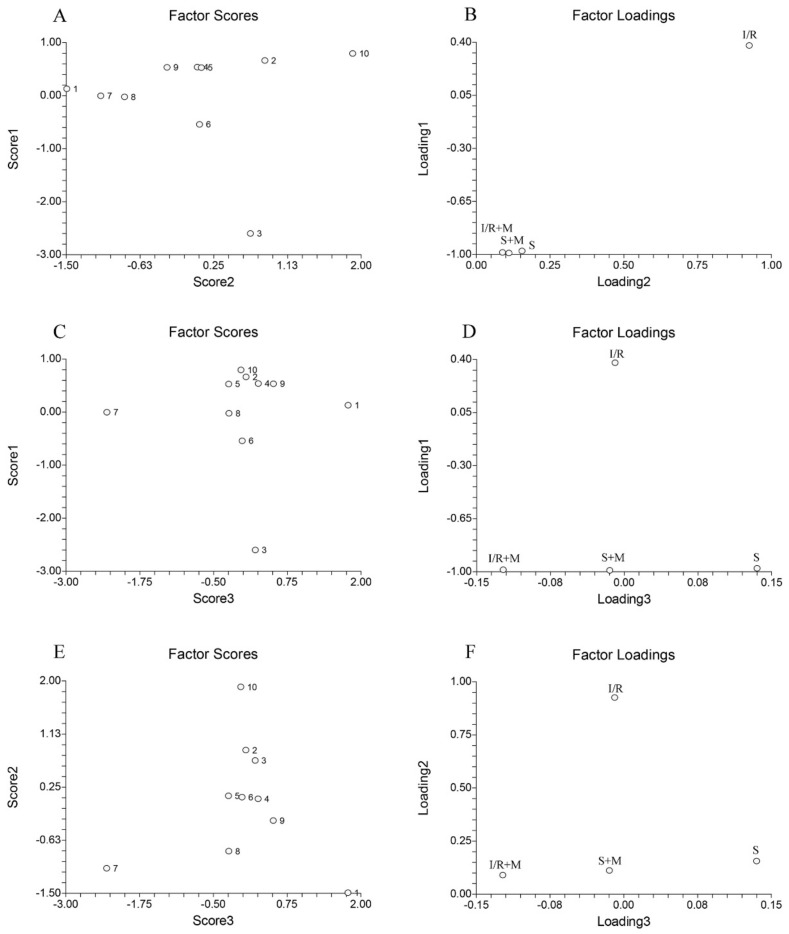
The score and the loading plots of kidney fatty acids principle component analysis. Group categories abbreviations: S—sham operated rat group; S + M—sham operated + meldonium rat group; I/R—ischemia/reperfusion rat group; I/R + M—ischemia/reperfusion + meldonium rat group. Number of animals per experimental group: *n* = 8. The numbers (1–10) on the score plots represent fatty acids: (1) C14:0; (2) C15:0; (3) C16:0; (4) C16:1; (5) C17:0; (6) C18:0; (7) C18:1 c + t; (8) C18:2 c + t; (9) C22:0; and (10) C22:6 + C22:1.

To explore potential similarities and differences between fatty acid profiles across all experimental groups the principal component analysis was applied (Figure 4). The data were organized in the 4 × 10 matrix, where rows represent four experimental groups and columns represent 10 fatty acids (alone or as the sum). The number of significant components (PCs) of this data set was determined by the percentage of modeled variance. The PCA model with three significant PCs was able to describe 99.85% of the data variance.

As can be seen from our results, the sum of C22:6 and C22:1 exhibits the highest positive value, whereas C16:0 exhibits the highest negative value (Figure 4A, numbers 10 and 3). Combining this data with the data from Figure 4Bs loading plot, it can be concluded that C16:0 has the highest influence on the separation of the I/R group relative to the S, S + M, and IR + M groups (Figure 4B). The greatest differences between C14:0 and C18:1 c + t (Figure 4C, numbers 1 and 7) is that the highest influence on the separation between the S and S + M groups (Figure 4D) had the C14:0 while the highest separation influence between the I/R + M and S + M groups (Figure 4D) had the C18:1 c + t. Finally, the greatest differences between C18:1 c + t and C22:6 relative to rests of fatty acids (Figure 4E, numbers 7 and 10) confirmed that the highest influence on the separation of the I/R group relative to the S, S + M, and IR + M groups (Figure 4F) had the C22:6, while the highest influence on separation between the I/R + M and S + M groups (Figure 4F) had the C18:1 c + t.

The small- and medium-chain-fatty acids are supposed to be able to enter the mitochondria by diffusion, whereas long chain fatty acids (C14–C20) require a transport system [58]. In mitochondria, this purpose serves carnitine palmitoyltransferase-1, the enzyme that meldonium inhibits, causing a reduction in long-chain fatty acids transporting from cytosol into mitochondria, as well as their redirection to peroxisomes. In contrast to mitochondria, the peroxisomal fatty acid transport system is realized by three classed of so-called ABC transporters. ABCD1 has the highest affinity to the C24:0–C26:0 fatty acids; ABCD2 has the highest affinity to the C22:0–C24:0 and C22:6; and ABCD3 has the highest affinity to the C20:5 and C16:0 fatty acids [24]. Our results showed that meldonium increases almost all fatty acids with the exception of C22:0 and C22:6 + C22:1 (Table 4), which suggests that meldonium restricts mitochondrial use of small and medium-chain-fatty acids, and that the unchanged concentration of C22:0 and C22:6 + C22:1 belongs to the peroxisomal metabolism. Concerning the effects of meldonium on I/R, it is known that euric acid (C22:1, number 10 on Figure 4A,C,E) represents an early sign of renal injury [59]. From this point, a 100-fold increase of C22:1 kidney concentration can be observed in rats subjected to I/R. This further confirms how extensive renal damage drastically decreases kidney concentration in animals from the I/R + M group. Alas, this confirms the protective effect of meldonium (Table 4).

## 3. Materials and Methods

### 3.1. Animals and Treatments

All animal procedures were performed in compliance with the ARRIVE guidelines and Directive 2010/63/EU. In accordance with the National legislation, all animal procedures were approved by the Veterinary Directorate of the Ministry of Agriculture and Forestry and Water Management, License number 323-07-07137/2018-05 (30.07.2019).

Wistar strain (*Rattus norvegicus*) male rats weighing 341.63 ± 4.95 g were used for the experiment. The animals were acclimated to 22 ± 1 °C and maintained under a 12 h light/dark period. The rats were randomly divided into four groups (eight animals per group) and housed two per cage for four weeks on ad libitum access to a standard diet (Veterinary Institute, Subotica, Serbia) and tap water (with or without meldonium). In total, we used 32 rats. Rat groups were as follows: animals that drank tap water for four weeks and then sham operated (S group); animals that drank tap water with meldonium for four weeks and then sham operated (S + M group); animals that drank tap water for four weeks and then I/R operated (I/R group); and animals that drank tap water with meldonium for four weeks and then I/R operated (I/R + M group).

Meldonium (3-(2,2,2-trimetilhidrazinijum) propionate; THP; MET-88) (manufacturer Shenzhen Calson Bio-Tech Co., Ltd., Shenzhen, China) was dissolved in tap water in concentrations ranging from 2–3 mg/ml. Depending on the water intake, meldonium concentrations in the water were adjusted weekly to achieve its consumption of around 300 mg/kg b.m./day. Based on the four-week measurement, the meldonium consumption was 302.01 ± 4.44 mg/kg b.m./day in sham operated animals and 301.42 ± 3.67 mg/kg b.m./day in I/R operated animals.

Body mass, body mass gain, and food and water intake were measured weekly throughout the experiment. The results were expressed as a time course of measured values and recalculated into the area under a curve (AUC) values.

### 3.2. Operative Procedures

Anesthetized rats were subjected to a non-recovery surgical procedure described elsewhere [9]. In brief, sodium thiopental (120 mg/kg, i.p.) was used for the induction of anesthesia. Animals were placed on their backs on a heating pad to provide constant body temperature of 37.5 ± 1 °C. The trachea, carotid artery, and jugular vein were separated from surrounding tissues and cannulated. The saline infusion was provided during the whole experiment (8 mL/kg/h before reperfusion, and 2 mL/kg/h during reperfusion) and anesthesia was maintained by sodium thiopental (10 mg/kg, i.v.). Rats were subsequently divided into two equal groups: one half was subjected to renal ischemia-reperfusion injury (I/R operated rats) while another half was followed up without any additional surgical procedure (sham operated rats).

Ischemia was produced using a reversible clamp that held both renal pedicles with the appropriate arterial clip for 45 min. At the beginning of reperfusion, clips were removed with big forceps and kidneys were subjected to reperfusion lasting for four hours. At the end of four-hour reperfusion, rats were euthanized using an overdose of thiopental sodium.

### 3.3. Sample Preparation

Blood and tissue samples were collected for further analysis immediately after euthanasia.

Blood was taken into the heparin-coated Eppendorf tubes directly from the intravenous catheter and inserted into the jugular vein, then incubated at room temperature for 45 min to allow clot formation. Clots were removed via centrifugation at 2000*× g* for 10 min in a refrigerated centrifuge. The resulting supernatant was immediately transferred into a clean polypropylene tube using a Pasteur pipette [60].

The liver, kidneys, and the adrenals of the rats were isolated and dissected out within 3 min, then placed in ice-cold 155 mmol NaCl and washed with the same solution. One part of the kidney was placed in formaldehyde for further histological analysis. The serum and the tissue samples for the rest of the analysis were stored at −80 °C until the analysis.

### 3.4. Biochemical Analysis

#### 3.4.1. Tissue Preparation

Tissue sample (50 mg) was mixed with 2 mL of ultra-pure water and pulverized with a tissue grinder. After extraction in an ultrasound bath at 0 °C, samples were centrifuged at 12,000 rpm. The supernatant was transferred in 10 mL normal flask and diluted with ultra-pure water to the mark. The sample solutions were kept at −80 °C till analysis.

#### 3.4.2. Tissue Lactic Acid Determination

The standard of lactic acid was purchased from Sigma Aldrich. Ion chromatography was used to assay the appearance and quantification of lactic acid. For this purpose, a Dionex ICS-3000 chromatographic setup was used, which consisted of a single pump, a conductivity detector (ASRS ULTRA II (4 mm) (P/N 061561), recycle mode), a eluent generator (potassium hydroxide (KOH) (P/N 058900)), and a Chromeleon^®^ Chromatography Workstation with Chromeleon 6.7.2 Chromatography Management software. The separation was performed on IonPac AS15 Analytical, 4 × 250 mm (P/N 053940) and IonPac AG15 Guard, 4 × 50 mm (P/N 053942) column. The mobile phase flow rate was set to 1000 ml/min, while the concentration of potassium hydroxide was changeable to achieve the following gradients: 0–15 min 10 mM KOH; 15–25 min 10–45 mM KOH; 25–26 min 45 mM KOH; 26–31 min 45–10 mM KOH; and 31–36 min 10 mM KOH. The column temperature was termostated at 30 °C. The conductivity cell temperature was 35 °C. The suppressor current was 134 mA. The backpressure was ~2200 psi.

#### 3.4.3. Tissue Glucose Determination

The standard glucose was purchased from Tokyo Chemical Industry, TCI, (Europe, Belgium). Sodium hydroxide and sodium acetate trihydrate were obtained from Merck (Darmstadt, Germany). All aqueous solutions were prepared using Ultrapure TKA deionized water. The standard solution of glucose was prepared in ultrapure water at 100 ng/mL concentration. Calibration standards were prepared from stock solution by dilution with ultrapure water. The quality control mixture used for monitoring instrument performance was prepared by diluting concentrations of 20 ng/mL. Chromatographic separations were performed using DIONEX ICS 3000 DP liquid chromatography system (Dionex, Sunnyvale, CA, USA) equipped with a quaternary gradient pump (Dionex, Sunnyvale, CA, USA) and a Carbo Pac^®^PA100 pellicular anion-exchange column (4 × 250 mm) (Dionex, Sunnyvale, CA, USA) at 30 °C. The mobile phase consisted of the following linear gradients (flow rate, 0.7 mL/min): 0–5 min, 15% A, 85% C; 5.0–5.1 min, 15% A, 2% B 83% C; 5.1–12 min, 15% A 2% B 83% C; 12–12.1 min, 15% A, 4% B, 81% C; 12.1–20 min 15% A, 4% B, 81% C; 20–20.1 min 20% A, 20% B 60% C; 20.1–30 min 20% A, 20% B 60% C; where A was 600 mM sodium hydroxide, B was 600 mM sodium acetate, and C was ultrapure water. Previously, the analysis system was preconditioned at 15% A and 85% C for 15 min. Each sample (25 µL) was injected with an ICS AS-DV 50 auto sampler (Dionex, Sunnyvale, CA, USA). The electrochemical detector consisted of gold as working and Ag/AgCl as reference electrode.

#### 3.4.4. Tissue Carnitine Determination

L-Carnitine was obtained from Sigma Aldrich. L-Carnitine (10 mg) was weighted in 10 mL of a normal flask diluted with ultra-pure water. Next, an ascending thin-layer chromatography was performed as an adsorbent on RP-18 silica (Art. 5559, Merck, Germany). The chromatograms were developed using an acetonitrile-water binary mixture with a ratio of 1/1 (*v*/*v*). A classical chromatographic chamber (Camag, Switzerland) was filled with 3 mL of mobile phase made by components of analytical grade of purity. The chamber was saturated with solvent for 30 min. All experiments were performed on ambient temperature. The plates were applied with CAMAG Linomat 5 with 2 µL aliquots of previous prepared and defrosted aqueous solutions. The plates were scanned using CAMAG TLC Scanner 3 at 260 nm. Chromatograms were analyzed using winCATS software version 1.4.2.8121.

#### 3.4.5. Tissue Fatty Acid Determination

The fatty acid analysis in the obtained samples was performed using Focus GC and a PolarisQ mass spectrometer (Thermo Fisher, Waltham, MA, USA). The carrier gas used in this experiment was helium (1 mL/min) and the injected volume of the sample was 1 µL. The initial temperature for the oven was 50 °C (1 min), then 25 °C/min to 200 °C, and immediately put to 3 °C/min to 230 °C (held for 18 min). The injector was in split mode (50:1) while the temperatures of injector, transfer line, and ion source were 250, 260, and 260 °C, respectively.

### 3.5. Determination of Oxidative Stress Biomarkers

The kidney samples were minced and homogenized in 10 volumes of 25 mmol/L sucrose. They contained 10 mmol/L Tris-HCl, had a pH of 7.5 at 1500 rpm, and used a Janke and Kunkel (Staufen, Germany) IKA-Werk Ultra-Turrax homogenizer at 4 °C. Homogenates were centrifuged at 4 °C at 100,000× *g* for 90 min and sonicated for 30 s at 10 kHz on ice (Bandeline Sonopuls HD 2070), followed by centrifugation in a Beckman ultracentrifuge at 100,000× *g* for 90 min at 4 °C. The obtained supernatants were used for biochemical analyses.

The activity of antioxidant defense enzymes was measured simultaneously in triplicate for each sample using a Shimadzu UV-1800 spectrophotometer and a temperature-controlled cuvette holder. The total SOD activity was measured in the supernatant by using the adrenaline method, which is based on the SOD capacity to inhibit adrenaline autoxidation to adrenochrome [61]. To determine MnSOD activity, an assay was performed after the pre-incubation with 8 mmol/L KCN. CuZnSOD activity was calculated as a difference between total SOD and MnSOD activities. SOD activity was expressed as U/g wet mass. CAT activity was determined as suggested by Claiborne [62] and expressed as µmol H_2_O_2_/min/g wet mass. The activity of GSH-Px was measured following the oxidation of nicotinamide adenine dinucleotide phosphate (NADPH) as a substrate at 340 nm with t-butyl hydroperoxide [63] and expressed in nmol NADPH/min/g wet mass. The activity of GR was evaluated as suggested by Glatzle et al. [64] and expressed in nmol NADPH/min/g wet mass. GST activity—toward 1-chloro-2,4-dinitro benzene (CDNB) as a substrate—was assayed according to Habig et al. [65] and expressed in nmol GSH/min/g wet mass. The GSH content was measured according to Griffith’s method [66], which was based on the sequential oxidation of GSH by 5,5′-dithiobis 2-nitrobenzoicacid (DTNB), then reduced by NADPH in the presence of GR. The GSH content was expressed as μmol GSH/g tissue. The concentration of SH groups was determined using DTNB according to the Ellman method [67] and expressed as nmol SH/g tissue. Lipid peroxide concentration was measured as thiobarbituric acid reactive substances (TBARS) in animal tissue. It was then assayed using the method found in Rehncrona et al. [68], which had thiobarbituric acid (TBA) as a reagent. In this reaction, the colored complex was formed and the absorbance was spectrophotometrically determined at 532 nm. Lipid peroxide concentration was expressed as nmol TBARS/g tissue. All chemicals used for the determination of oxidative stress parameters were SIGMA (St. Louis, MO, USA) products.

### 3.6. Determination of Vitamin C Concentration

The serum, liver, and adrenal concentration of AA and DHA were measured according to the method established by Nováková et al. [69]. L-Ascorbic acid, phosphoric acid solution (49–51%), meta-phosphoric acid (MPA), sodium phosphate monobasic dihydrate, and dithiothreitol (DTT) were purchased from Sigma-Aldrich. Purified water was obtained via a BlueClearRO600P reverse osmosis water cleaner system with an integrated BlueSoft07-MB mixed bed salt remover (Euro-Clear Ltd., Hungary).

A stock standard solution of L-Ascorbic acid (1 mg/mL) was prepared in 10% MPA and kept at −20 °C. Standard solutions were prepared by diluting the stock standard solution in 10% MPA.

The phosphate buffer (160 mM, pH 3.0) was used as the mobile phase. Sodium phosphate monobasic dihydrate (26 g) was accurately weighed, dissolved in approximately 800 mL of water, and the pH was adjusted to a value of 3.0 with a phosphoric acid solution (49–51%). The resulting solution was made up to 1 l with water.

Tissue samples were homogenized in 10% MPA (1 mg:10 µL), using an Ultra-Turrax homogenizer, and sonicated (3 × 10 s). Plasma samples were added to a 10% MPA (9:1, *v*:*v*). All samples were centrifuged (30 min, 18,000 rpm, 4 °C). Supernatants were used for the analysis of native AA concentration. For the analysis of TVC concentration, 100µL of the above supernatant was treated with 300 µL DTT solution (2.5 mg/mL in phosphate buffer) for 30 min at 4 °C for complete conversion of DHA to AA. The mixture was then re-acidified with 200 µL of 10% MPA and transferred to the autosampler unit.

The data were obtained using the same HPLC system as described earlier. The following chromatographic conditions were obtained after the method optimization. The mobile phase that consisted of the phosphate buffer (160 mM, pH 3.0) was pumped over 10 min in an isocratic flow of 800 µL/min. The applied potential for electrochemical measurements was 600 mV and the separation temperature was 25 °C. The 20 µL of samples and standard solutions were applied to the system. The concentration of AA and DHA were expressed as µg/mg tissue and µg/mL serum.

### 3.7. Determination of Catecholamine Concentration

The concentration of adrenaline (AD) and noradrenaline (NA) in the adrenal glands was performed according to the method of Stefanovic et al. [70]. Ethylene glycol tetra-acetic acid (EGTA), 5-hydroxytryptamine (5-HT), DL-Noradrenaline hydrochloride (NA), perchloric acid (70%), and magnesium chloride (MgCl_2_ × 5H_2_O) were purchased from Sigma-Aldrich. Ammonium formate was supplied by Fisher Scientific, formic acid (49–51%) by Fluka, and methanol by J.T. Baker. Purified water was obtained via a BlueClearRO600P reverse osmosis water cleaner system with an integrated BlueSoft07-MB mixed bed salt remover (Euro-Clear Ltd., Gönyű, Hungary).

Stock standard solutions of catecholamines (1 mg/mL) were prepared in methanol and kept at –20 °C. Standard solutions were prepared by diluting the stock standard solution in DEPROT (2% EGTA; 0.1 N HClO_4_; 0.2% MgCl_2_).

The ammonium formate buffer (100 mM, pH 3.6) was used as one of the mobile phase components. Ammonium formate (6.3 g) was accurately weighed and dissolved in approximately 800 ml of water. Then, the pH was adjusted to the value of 3.6 with formic acid. The resulting solution was made up to 1 l with water.

Tissue samples were homogenized in DEPROT (1 mg:10 µL) using an Ultra-Turrax homogenizer. They were then sonicated (3 × 10 s) and centrifuged (30 min, 18,000 rpm, 4 °C). Supernatants were transferred in separate tubes and placed in the HPLC system’s autosampler.

We obtained the data using a Thermo Scientific (Dionex UltiMate 3000) HPLC system that consisted of a degasser unit, binary pump, autosampler, column compartment, and RS electrochemical detector equipped with a glassy carbon working electrode. An Acclaim Polar Advantage II, C18 (3 µm) HPLC column (ThermoScientific, Waltham, MA, USA) was used. Instrument control and data acquisition were carried out using a Chromeleon7 Chromatography Data System (ThermoScientific).

The following chromatographic conditions were obtained after the method optimization. The mobile phase that had an ammonium formate buffer (100 mM, pH 3.6) as an A solution and methanol as a B solution was pumped at a flow rate of 500 µL/min with the following gradient. The run was started with a mobile phase consisting of 98% A and 2% B solution. Starting from the ninth minute of the run, the B solution rose to reach 80% in the 13th min. Starting from the 18th min until the end of the run (25th min), the column was re-equilibrated with the initial mixture of each mobile phase solution (2% of A and 98% of B solution). The potential for electrochemical measurements was 850 mV; the separation temperature was 25 °C. Next, 40 µL worth of samples and standard solutions were applied into the system. The concentration of catecholamine was expressed as µg/mg tissue.

### 3.8. Kidney Homogenate Preparation

To prepare for the kidney homogenate, tissue was homogenized in an ice-cold homogenization buffer (250 mM sucrose, 10 mM Tris-HCl, pH 7.6, 1 mM EDTA), which was supplemented with 1× phosphatase inhibitor Mix I and 1× protease inhibitor Mix G (Serva). After sonication, the homogenate was centrifuged at 100 000× *g* in a Beckmann rotor Ti 50 (Beckman Coulter Inc., Brea, CA 92821, USA) for 90 min. The resulting supernatant was aliquoted, snap-frozen in liquid nitrogen, and stored at −80 °C.

### 3.9. Western Immunoblot Analysis

Protein samples of serum (1 µL) or kidney homogenates (50 µg) were separated by 12% SDS-PAGE and transferred onto polyvinylidene difluoride (PVDF) membranes (Hybond-P, Amersham Pharmacia Biotech, Little Chalfont, United Kingdom). These samples were then blocked in a solution (0.2% Tween 20, 50 mM TrisHCl pH 7.6, 150 mM NaCl) containing 5% non-fat condensed milk. After the protein transfer, PVDF membranes were incubated with a primary antibody for 1.5 h at room temperature. Western immunoblot analysis was performed using Abcam rabbit polyclonal HMGB1 (ab 18256), Nrf2 (ab 92946), HO-1 (ab 13243), β actin (ab 8227), Bax (2772S), Bcl-2 (2876S) antibodies, Santa Cruz Biotechnology rabbit polyclonal MnSOD/SOD-2 (sc 30080), p38 (sc 535), p-p38 (sc 7975-R), Akt 1/2/3 (H-136), pAkt1/2/3 (ser 473)-R antibodies, and a goat polyclonal anti-HMGB1 antibody for serum analysis (K12). The blots were probed with horseradish peroxidase-conjugated secondary Santa Cruz Biotechnology bovine anti-rabbit IgG pr (sc 2379), bovine anti-goat IgG (sc 2378), and Abcam goat anti-rabbit IgG (ab 97051) antibodies. Immunoreactive bands were identified using an enhanced chemiluminescence (ECL) detection system (Santa Cruz Biotechnology, Santa Cruz, CA, USA) according to the manufacturer′s instructions. The blots were scanned and the intensities of the signals were quantified using TotalLab (Phoretix, Newcastle Upon Tyne, UK) electrophoresis software (ver.1.10). Actin was used as a loading control for the quantification. For re-probing, membranes were incubated in 2% SDS, 100 mM β-mercaptoethanol, and 62.5 mM Tris-HCl pH 6.8 for 35 min at 50 °C, then rinsed three times, blocked, and probed again with another antibody. All immunoblot analyses were obtained from at least three independent experiments.

### 3.10. Histology Analysis

Kidney samples were collected and fixated in 4% formaldehyde solution. After fixation, the samples were dehydrated in a series of increasing ethanol solutions (70%, 96%, and 100%, respectively) followed by an immersion in a clearing agent (xylene). The tissue samples were embedded into paraffin wax and cut into 5 µm thick sections. A minimum of 10 fields for each kidney slide were examined using a Leica DM LS 2 type 11020518016 microscope (Leica, Wentzler, Germany). The histological analysis evaluated tubular necrosis, interstitial oedema, loss of brush border, and casts formation.

### 3.11. Statistical Analysis

Where appropriate, the results were expressed graphically as time-course curves, which were subsequently recalculated into area under a curve (AUC) values. The single time-point measurements were presented as mean ± standard errors of mean. AUC values were presented as the percentage of controls. Data were checked for normality using Lilliefors and Kolmogorov–Smirnov tests. Differences in investigated parameters between groups were calculated using one-way ANOVA. When significant differences were found, pairwise comparisons were performed using Holm-Sidak post hoc tests. The statistical package SIGMAPLOT was used for all the analyses and graphical presentations, except for the principal component analysis (PCA), which was performed using the NCSS 2004 software package. The level of statistical significance was defined as *p* < 0.05.

## 4. Conclusions

Table 5 summarizes the main effects of meldonium and I/R in our experiment. According to the presented results, it can be concluded that meldonium protects the kidney against I/R-induced injury in many ways. This includes both anti-apoptotic and anti-necrotic effects, as well as anti-inflammatory and antioxidative ones. In the case of vitamin C, the antioxidative effect of meldonium does not appear to be limited to the kidney, but instead extends to the surrounding organs. We also proved that meldonium increases adrenal concentration of noradrenaline, but this does not yet have an adequate explanation. Still, this result represents an original contribution to the study of the effects of meldonium, just like the results suggesting that meldonium has the ability to act as an anti-obesity agent. Our results of kidney lipidomics are also a novelty in the meldonium study area. Given that the literature in this field is very scarce, we will direct our future experiments toward clarifying the link between the meldonium and its effects on lipidomics.

## Figures and Tables

**Table 1:** Kidney carnitine, glucose, and lactic acid concentration (μg/g wet tissue mass). Group categories abbreviations: S—sham operated rat group; S + M—sham operated + meldonium rat group; I/R—ischemia/reperfusion rat group; I/R + M—ischemia/reperfusion + meldonium rat group. Number of animals per experimental group: *n* = 8. The data are given as mean ± standard error. Minimal significant level: *p* < 0.05. Significantly different: **^a^** in respect to S; **^b^** in respect to S + M; **^c^** in respect to I/R group.

Experiment Groups	S	S + M	I/R	I/R + M
Carnitine	486.39 ± 34.02	148.66 ± 7.79 **^a^**	471.49 ± 22.7 **^b^**	385.03 ± 13.43 **^abc^**
Glucose	4.16 ± 0.27	1.85 ± 0.15 **^a^**	3.88 ± 0.35 **^b^**	2.28 ± 0.20 **^ac^**
Lactic acid	319.34 ± 25.90	11.83 ± 0.99 **^a^**	1164.07 ± 150.65 **^ab^**	685.54 ± 60.56 **^abc^**

**Table 2:** Activities of kidney copper-zinc superoxide dismutase (CuZnSOD, U/g wet mass), manganese superoxide dismutase (MnSOD, U/g wet mass), catalase (CAT, μmol H_2_O_2_/min/g wet mass), glutathione peroxidase (GSH-Px, μmol NADPH/min/g wet mass), glutathione reductase (GR, nmol NADPH/min/g wet mass), and glutathione S-transferase (GST, nmol GSH/min/g wet mass), as well as kidney concentrations of sulfhydryl groups (SH, nmol/g tissue) and lipid peroxides (TBARS, nmol/g tissue). Group categories abbreviations: S—sham operated rat group; S + M—sham operated + meldonium rat group; I/R—ischemia/reperfusion rat group; I/R + M—ischemia/reperfusion + meldonium rat group. Number of animals per experimental group: *n* = 8. The data are given as mean ± standard error. Minimal significant level: *p* < 0.05. Significantly different: **^a^** in respect to S; **^b^** in respect to S + M; **^c^** in respect to I/R group.

Experiment Groups	S	S + M	I/R	I/R + M
CuZnSOD	2183.12 ± 126.99	1531.98 ± 45.87 ^a^	2072.75 ± 93.81 ^b^	1231.84 ± 117.88 ^ac^
MnSOD	305.22 ± 22.94	644.35 ± 43.23 ^a^	250.98 ± 6.07 ^ab^	367.60 ± 32.99 ^bc^
CAT	15,323.99 ± 494.06	15,569.04 ± 440.31	12,832.91 ± 348.47 ^ab^	12,543.406 ± 512.92 ^ab^
GSH-Px	41.69 ± 2.22	43.27 ± 2.36	42.94 ± 3.034	24.80 ± 0.97 ^abc^
GR	16,495.17 ± 493.63	16,495.17 ± 696.70	16,495.17 ± 898.39	13,216.03 ± 277.42 ^abc^
GST	25,328.13 ± 727.06	25,496.88 ± 878.78	24,042.19 ± 837.08	25,667.19 ± 1002.05
SH	3955.56 ± 215.29	5240.88 ± 357.92 ^a^	3182.59 ± 105.86 ^ab^	4496.62 ± 559.32 ^c^
TBARS	1.24 ± 0.04	1.23 ± 0.03	1.44 ± 0.03 ^ab^	1.26 ± 0.04 ^c^

**Table 3:** The ascorbic/dehydroascorbic acid ratio (AA/DHA, % of sham) in serum, liver, adrenal gland, and kidney, as well as adrenal noradrenaline (NA) and adrenaline (AD) content (µg/mg tissue). Group categories abbreviations: S—sham operated rat group; S + M—sham operated + meldonium rat group; I/R—ischemia/reperfusion rat group; I/R + M—ischemia/reperfusion + meldonium rat group. Number of animals per experimental group: *n* = 8. The data are given as mean ± standard error. Minimal significant level: *p* < 0.05. Significantly different: **^a^** in respect to S; **^b^** in respect to S + M; **^c^** in respect to I/R group.

Experiment Groups	S	S + M	I/R	I/R + M
Kidney AA/DHA	100.00 ± 8.76	134.93 ± 7.48 **^a^**	64.97 ± 6.77 **^ab^**	138.73 ± 7.83 **^ac^**
Liver AA/DHA	100.00 ± 8.57	181.28 ± 12.15 **^a^**	71.51 ± 6.64 **^ab^**	124.60 ± 10.12 **^bc^**
Serum AA/DHA	100.00 ± 4.44	197.26 ± 17.02 **^a^**	59.99 ± 3.37 **^ab^**	91.81 ± 7.25 **^bc^**
Adrenal AA/DHA	100.00 ± 5.60	309.07 ± 12.97 **^a^**	69.12 ± 4.83 **^ab^**	221.09 ± 12.95 **^abc^**
Adrenal NA	0.340 ± 0.011	2.185 ± 0.105 **^a^**	0.155 ± 0.01 **^ab^**	0.462 ± 0.029 **^abc^**
Adrenal AD	1.244 ± 0.228	1.068 ± 0.147	1.485 ± 0.346	1.115 ± 0.238

**Table 4:** Kidney fatty acid concentration (µg/g tissue). Group categories abbreviations: S—sham operated rat group; S + M—sham operated + meldonium rat group; I/R—ischemia/reperfusion rat group; I/R + M—ischemia/reperfusion + meldonium rat group. Number of animals per experimental group: *n* = 8. The data are given as mean ± standard error. Minimal significant level: *p* < 0.05. Significantly different: **^a^** in respect to S; **^b^** in respect to S + M; **^c^** in respect to I/R group.

Experiment Groups	S	S + M	I/R	I/R + M
C14:0	16.33 ± 2.67	42.00 ± 11.66 **^a^**	6.10 ± 1.55 **^ab^**	23.50 ± 6.09 **^bc^**
C15:0	0.11 ± 0.02	1.29 ± 0.36 **^a^**	85.08 ± 21.56 **^ab^**	7.23 ± 1.87 **^abc^**
C16:0	138.75 ± 22.66	460.83 ± 127.95 **^a^**	39.06 ± 9.90 **^ab^**	668.69 ± 173.36 **^ac^**
C16:1	0.09 ± 0.02	14.38 ± 3.99 **^a^**	58.96 ± 12.12 **^ab^**	5.18 ± 1.34 **^abc^**
C17:0	0.09 ± 0.02	0.34 ± 0.0 **^a^**	60.45 ± 14.94 **^ab^**	35.66 ± 9.24 **^ab^**
C18:0	47.32 ± 7.73	148.22 ± 41.15 **^a^**	46.34 ± 15.32 **^b^**	251.69 ± 65.25 **^ac^**
C18:1 c + t	0.23 ± 0.04	78.19 ± 21.71 **^a^**	17.99 ± 11.74 **^ab^**	160.71 ± 41.67 **^ac^**
C18:2 c + t	18.78 ± 3.07	53.77 ± 14.93 **^a^**	25.70 ± 4.56 **^ab^**	140.53 ± 36.43 **^abc^**
C22:0	0.46 ± 0.07	0.51 ± 0.14	47.85 ± 12.12 **^ab^**	0.04 ± 0.01 **^abc^**
C22:6 + C22:1	0.80 ± 0.13	1.11 ± 0.31	118.57 ± 30.04 **^ab^**	0.11 ± 0.01 **^abc^**

**Table 5:** Summarized effects of meldonium and I/R on the kidney carnitine, glucose, and lactic acid concentration; the protein expression levels of serum HMGB1 and kidney MnSOD; Bax, Bcl-2, HMGB1, Nrf2, HO-1, and total and phosphorylated p38/Akt; the kidney activities of copper-zinc superoxide dismutase (CuZnSOD), manganese superoxide dismutase (MnSOD), catalase (CAT), glutathione peroxidase (GSH-Px), glutathione reductase (GR), and glutathione S-transferase (GST); the kidney concentrations of sulfhydryl groups (SH) and lipid peroxides (TBARS); the serum, liver, adrenal gland, and kidney ascorbic/dehydroascorbic acid ratio (AA/DHA); the adrenal noradrenaline (NA) and adrenaline (AD) content; and the kidney fatty acids concentration. Group categories abbreviations: S—sham operated rat group; S + M—sham operated + meldonium rat group; I/R—ischemia/reperfusion rat group; I/R + M—ischemia/reperfusion + meldonium rat group. Up arrow — an increase; Down arrow — a decrease.

Experiment Groups	S + M vs. S	I/R vs. S	I/R + M vs. I/R
Carnitine	↓	none	↓
Glucose	↓	none	↓
Lactic acid	↓	↑	↓
Bax/Bcl2 ratio	none	↑	↓
Serum HMGB1	none	↑	↓
Kidney HMGB1	none	↑	↓
MnSOD expression	↑	↓	↑
pp38/p38 ratio	↑	↓	↑
pAkt/Akt ratio	↑	↓	↑
Nrf2	none	↓	↑
HO1	↑	↓	↑
MnSOD	↑	↓	↑
CuZnSOD	↓	none	↓
CAT	none	↓	none
GSH-Px	none	none	↓
GR	none	none	↓
GST	none	none	none
SH	↑	↓	↑
TBARS	none	↑	↓
Kidney AA/DHA	↑	↓	↑
Liver AA/DHA	↑	↓	↑
Serum AA/DHA	↑	↓	↑
Adrenal AA/DHA	↑	↓	↑
Adrenal NA	↑	↓	↑
Adrenal AD	none	none	none
C14:0	↑	↓	↑
C15:0	↑	↑	↓
C16:0	↑	↓	↑
C16:1	↑	↑	↓
C17:0	↑	↑	↓
C18:0	↑	none	↑
C18:1 c + t	↑	↑	↑
C18:2 c + t	↑	↑	↑
C22:0	none	↑	↓
C22:6 + C22:1	none	↑	↓
